# Non-metastatic causes of multiple pulmonary nodules

**DOI:** 10.1186/s13244-024-01856-9

**Published:** 2024-11-29

**Authors:** Esra Akçiçek, Gamze Durhan, Selin Ardalı Düzgün, Olcay Kurtulan, Meltem Gülsün Akpınar, Figen Demirkazık, Orhan Macit Arıyürek

**Affiliations:** 1 Department of Radiology, Hacettepe University Faculty of Medicine, Ankara, Turkey; 2 Department of Pathology, Hacettepe University Faculty of Medicine, Ankara, Turkey

**Keywords:** Multiple pulmonary nodules, Metastasis, Computed tomography, Diagnostic imaging

## Abstract

**Abstract:**

Various processes, including benign or malignant (mostly metastasis) processes, contribute to the occurrence of multiple pulmonary nodules. For differential diagnosis, metastasis must be excluded as an etiological factor in patients who have multiple pulmonary nodules with a known primary malignancy. However, differential diagnosis of multiple pulmonary nodules caused by benign diseases and malignant processes is challenging. Multiple pulmonary nodules resulting from metastasis may mimic those resulting from infections, inflammatory processes, and rare benign diseases. Some rare diseases, such as pulmonary sclerosing pneumocytoma and pulmonary epithelioid hemangioendothelioma, or common diseases with a rare presentation of multiple nodules must be considered in the differential diagnosis of metastasis. In addition to the clinical and laboratory findings, radiological features are crucial for differential diagnosis. The size, density, location, and border characteristics (well-defined or poorly defined) of pulmonary nodules, as well as their internal structure (solid, subsolid, or ground glass nodule), growth rate during follow-up, and associated pulmonary and extrapulmonary findings are important for differential diagnosis along with clinical and laboratory data. This article summarizes the general features and imaging findings of these diseases, which less frequently present with multiple pulmonary nodules, and the clues that can be used to distinguish these diseases from metastasis.

**Critical relevance statement:**

The radiological features, clinical findings, and temporal changes during follow-up are important in distinguishing non-metastatic causes of multiple pulmonary nodules from metastatic causes and guiding diagnosis and early treatment, especially in patients with primary malignancy.

**Key Points:**

- Multiple pulmonary nodules have a wide range of etiologies, including metastatic disease.
- Metastasis as an etiology must be excluded in patients with multiple pulmonary nodules.
- Correlation of radiological findings (nodule size, position, and associated findings) with clinical history is crucial for differential diagnosis.

**Graphical Abstract:**

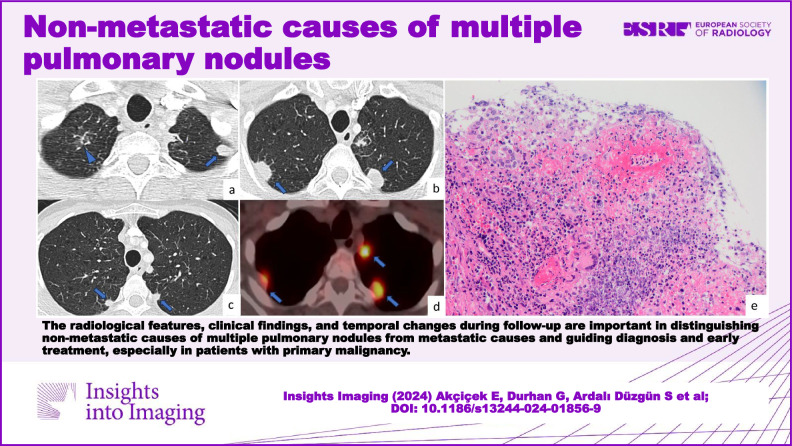

## Introduction

The morphology of multiple pulmonary nodules may range from a diffuse micronodular pattern to well-defined large nodules. Computed tomography (CT) is the first examination for evaluating multiple pulmonary nodules or masses. The presence of multiple, round pulmonary nodules in a patient with a primary malignancy in the presence or absence of fluorodeoxyglucose (FDG) uptake on positron emission tomography-CT (PET-CT) scans should be considered highly suspicious for metastasis. Multiple pulmonary nodules are frequently observed due to metastatic causes. However, several other diseases, including infections, inflammatory processes, and rare benign diseases, must be considered for differential diagnosis. Some rare diseases, such as pulmonary sclerosing pneumocytoma (PSP), or common diseases with a rare presentation of multiple nodules can also be considered in the differential diagnosis of metastasis. Classifying nodules according to their different radiologic characteristics, clinical findings, and temporal changes during follow-up can contribute to the success of differential diagnoses. This pictorial review discusses non-metastatic causes of multiple pulmonary nodules (Table 1) that may mimic metastatic causes. This article aimed to provide a detailed overview of various non-metastatic causes of pulmonary nodularity with a focus on imaging characteristics and temporal changes.

**Table 1:** The characteristics of the diseases causing multiple pulmonary nodules that can be used in differential diagnosis

	Clinical findings and patient characteristics	Radiological findings	Pathological findings	Microbiological/serological findings
Tuberculosis	Asymptomatic, or fever, malaise, and weight loss Immuncompromised	Multiple bilateral macronodules Upper lobe predilection Calcification +/− Cavitation +/−	Necrotizing granulomatous inflammation	No gold-standard test Culture of *M. tuberculosis* Some molecular tests for *M. tuberculosis* antigens Tuberculin skin test (TST)
Fungal infection	Dyspnea, fatigue, cough, fever and hemoptysis	Consolidation or multiple nodules Upper lobe predilection Solid/subsolid Halo sign +/−	Necrotizing or non-necrotizing granulomas, cellular interstitial pneumonia Intra-alveolar foamy material	Serum or BAL galactomannan PCR on serum, blood or lung biopsy sample
Pulmonary hydatid cyst	Asymptomatic, or cough, chest pain, fever, fatigue, and weight loss	Low-density, homogeneous, well-defined, round, or oval nodules or masses Check up on liver for coexisting liver HC	Biopsy should be avoided due to the risk of rupture and contamination	Serological evidence of hydatic cyst (hemagglutination (IHA), immunoblotting, enzyme-linked immunosorbent assay (ELISA), etc.)
Sarcoidosis	Nonspecific (cough and dyspnea) Skin disease such as erythema nodosum	Multiple, bilateral pulmonary perilymphatic micronodules Upper and middle lobe predilection Hilar and mediastinal lymphadenopathies	Non-infectious, non-caseating granulomas	Biomarkers like angiotensin converting enzyme (ACE), soluble interleukin-2 receptor (sIL-2R) (suboptimal sensitivity and specificity)
Pneumoconiosis	Asymptomatic, or shortness of breath, nonproductive cough Occupational and/or environmental history	Well-defined small perilymphatic nodules Upper lobe and posterior region predilection Hilar and mediastinal lymphadenopathies (+/− calcification)	Fibroinflammatory pattern	-
Rheumatoid pulmonary nodules	Mostly asymptomatic, or clinical findings of systemic RA	Single or multiple nodules Middle and upper zones Peripheral and subpleural location Cavitation +/−	Central fibrinoid necrosis surrounded by epithelioid cells, lymphocytes, plasma cells, and fibroblasts	Rheumatoid factor (RF) or anti-citrullinated protein antibodies (ACPA)
Granulomatosis with polyangiitis	Cough, mild dyspnea, hemoptysis and chest pain	Multiple, bilateral pulmonary nodules No specific location Range in size (up to 10 cm) Cavitation +/−	Necrosis, granulomatous inflammation, and vasculitis	Serum c-antineutrophil antibodies against protease 3 in cytoplasmic granules (c-ANCA)
Cryptogenic organizing pneumonia	Flu-like symptoms (fever, cough, fatigue) or shortness of breath	Solitary or multiple nodules or masses Well-defined, poorly defined Solid, partially solid, or ground-glass No specific distribution Reverse halo (atoll) sign +/−	Alveolar epithelial damage and leakage of plasma proteins into the alveolar space and recruitment of inflammatory cells	-
Pulmonary Langerhans cell histiocytosis	Non-productive cough, dyspnea, or fever Smoking history	Multiple bronchiolocentric, symmetrical, upper lobe-predominant nodules Sparing both lung bases and costophrenic angles	Clustering of Langerhans cells within the small airways	-
Nodular pulmonary amyloidosis	Asymptomatic, or wheeze, cough, and recurrent pneumonia Presence of Sjogren’s syndrome	Calcified or cavitary nodular opacities (with sharp, smooth, and lobulated contours) Lower lobes at the periphery	Abnormal accumulation of amyloid fibrils in the extracellular space of the lung tissue	-
Pulmonary epithelioid hemangioendothelioma	Mostly asymptomatic, or chest tightness and shortness of breath	Bilateral randomly distributed multiple parenchymal nodules Located near medium-sized vessels and bronchi	Epithelioid cells with abundant eosinophilic cytoplasm found within a fibromyxoid stroma	-
Minute pulmonary meningothelial-like nodules	Mostly asymptomatic, or dry cough and/or dyspnea	Small, randomly distributed, solid or subsolid multiple nodules, or as tiny rings with central lung attenuation (Cheerio sign)	Proliferation of meningothelial cells within the lung tissue	-
Diffuse idiopathic pulmonary neuroendocrine cell hyperplasia (Pulmonary Tumorlets)	Asymptomatic, or dyspnea and chronic cough.	Multiple bronchocentric, well-circumscribed solid nodules located lower zones Air trapping	Proliferation of neuroendocrine cells forming nodular or linear clusters	-
Pulmonary sclerosing pneumocytoma	Mostly asymptomatic	Well-defined, round, or oval, juxta-pleural nodules with strong, homogeneous enhancement	Cuboidal surface cells, and round stromal cells	-

## Infectious diseases

### Tuberculosis (TB)

TB, an infectious disease caused by *Mycobacterium tuberculosis*, is a major health problem in developing countries with poor public health facilities. In TB, the lung is the most frequently affected organ. The clinical and radiological manifestations of pulmonary TB depend on primary and post-primary infections. Patients with primary or post-primary TB are often asymptomatic. Symptomatic patients exhibit constitutional symptoms, such as fever, malaise, and weight loss. Additionally, progressive primary TB can affect immunocompromised patients [1]. The characteristic histopathological feature of tissues infected with *M. tuberculosis* is the presence of necrotizing granulomatous inflammation, which comprises clusters of epithelioid histiocytes surrounding a central necrotic area. Additionally, variable numbers of multinucleated giant cells and lymphocytes may be present within the granuloma [2].

**Fig. 1:**
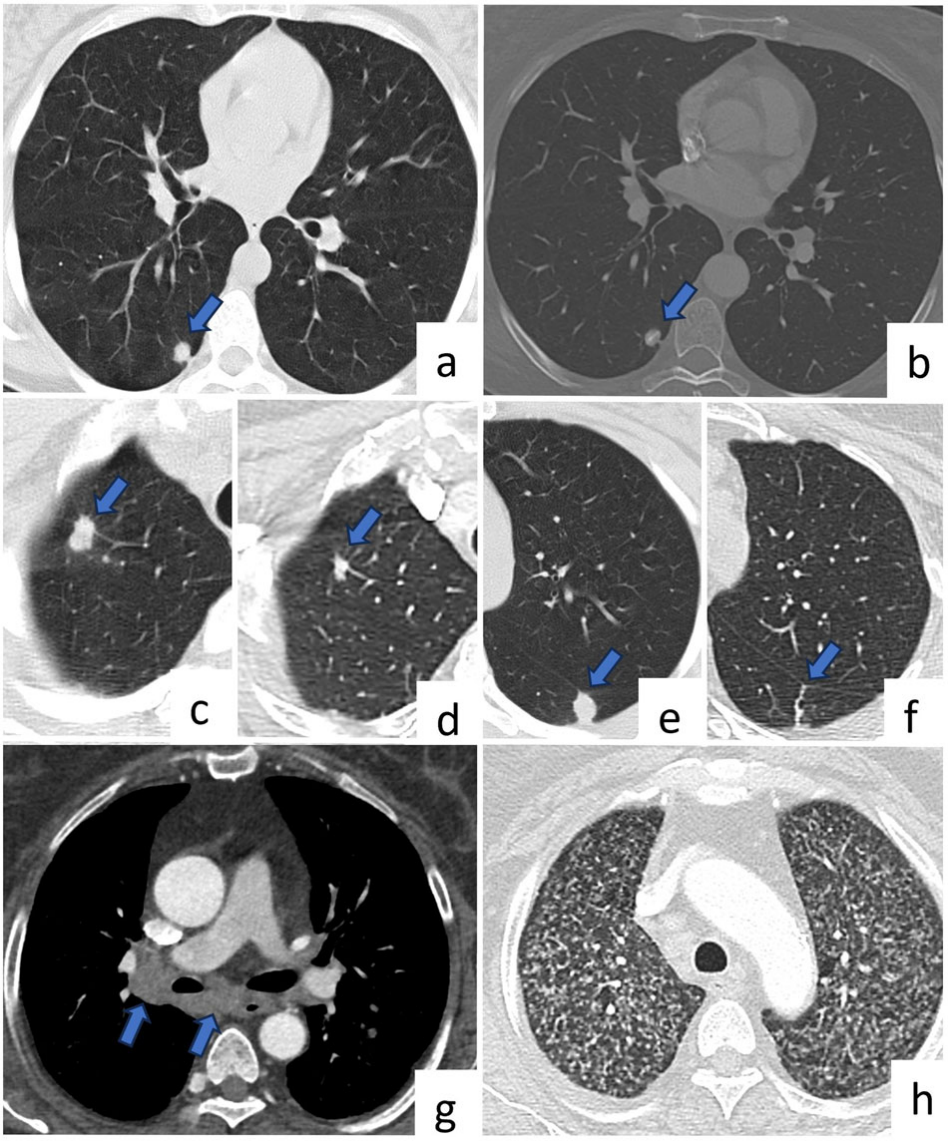
Tuberculosis. A 57-year-old patient with diabetes has multiple lung nodules incidentally detected. Axial CT images (**a**–**f**) show incidentally detected multiple lung nodules (arrows) diagnosed as pulmonary tuberculosis. On axial CT image (bone window) (**b**), a calcified nodule is seen. Axial CT images obtained between 2006 (**c**, **e**) and 2022 (**d**, **f**) show that there is a partial size reduction of the nodule in the right upper lobe (**c**, **d**), and the nodule in the left lower lobe has turned into a fibrotic band (**e**, **f**) after antituberculosis treatment. In a 71-year-old female patient diagnosed with miliary tuberculosis, axial CT image reveals right hilar and subcarinal lymphadenopathy (**g**, arrows), along with widespread and numerous micronodules in both lungs (**h**)

Radiological features depend on the type of infection. In primary TB, the first focus can occur anywhere in the lung and can manifest as a millimetric focus or as a patchy lobar consolidation with no specific appearance. Cavitation is uncommon in primary TB. Post-primary TB often presents as heterogeneous, poorly circumscribed, and often cavitating opacity. Cavity size may reach up to several centimeters (10–30%). The typical locations of these lesions are apical and posterior segments of the upper lobes and superior segments of the lower lobes. Bronchogenic dissemination is a complication of tuberculous cavitation and usually presents as numerous ill-defined micronodules, mostly located in the lower lung lobes [3]. Some patients can have miliary TB, which presents as multiple uniform micronodules (1–4 mm) (Fig. 1).

Tuberculoma, which is a rare mass or nodule caused by TB, is often seen as a single nodule in the upper lobe and may contain calcification (Fig. 1), usually appearing as a sharp-edged and rounded opacity [4]. Multiple bilateral noncavitated pulmonary macronodules of tuberculomas are highly unusual in pulmonary TB [3]. This presentation is mostly seen in patients with impaired immune system age-affinity. Thus, the presence of multiple larger pulmonary nodules is considered metastases, especially in patients with primary cancer. Although TB with the presentation of multiple pulmonary nodules is rare, it should be considered during differential diagnosis in developing countries and in patients with impaired immune system. The presence of centrilobular or satellite micronodules around multiple larger nodules and the presence of nodules in typical locations, such as the apical and posterior segments of the upper lobes or superior segments of the lower lobes, are the major radiological findings for the differential diagnosis of TB nodules. Concomitant cavitation or calcification within nodules and clinical findings may also be useful for correct diagnosis.

### Fungal infection

Fungal infection is a common condition in all populations. Lung involvement in fungal infection is common. The presentations of fungal infection vary between immunocompetent and immunocompromised patients. Various fungi are the causative agents for pulmonary fungal infections. Pulmonary aspergillosis, candidiasis, histoplasmosis, blastomycosis, cryptococcosis, and mucormycosis are the most frequent fungal diseases. The most common fungal disease in immunocompromised individuals is pulmonary aspergillosis. The mortality rates of invasive pulmonary aspergillosis are high in immunocompromised patients. The clinical symptoms of these infections are nonspecific and can include dyspnea, fatigue, cough, and even hemoptysis. The diagnosis of the infection may be delayed due to the low sensitivity of laboratory tests and nonspecific symptoms, requiring a biopsy analysis. The immunological competence of the patient is correlated with the severity of fungal infections in pathological samples, which are characterized by necrotizing or non-necrotizing granulomas, cellular interstitial pneumonia, intra-alveolar foamy material, or minimal inflammatory alterations [5].

**Fig. 2:**
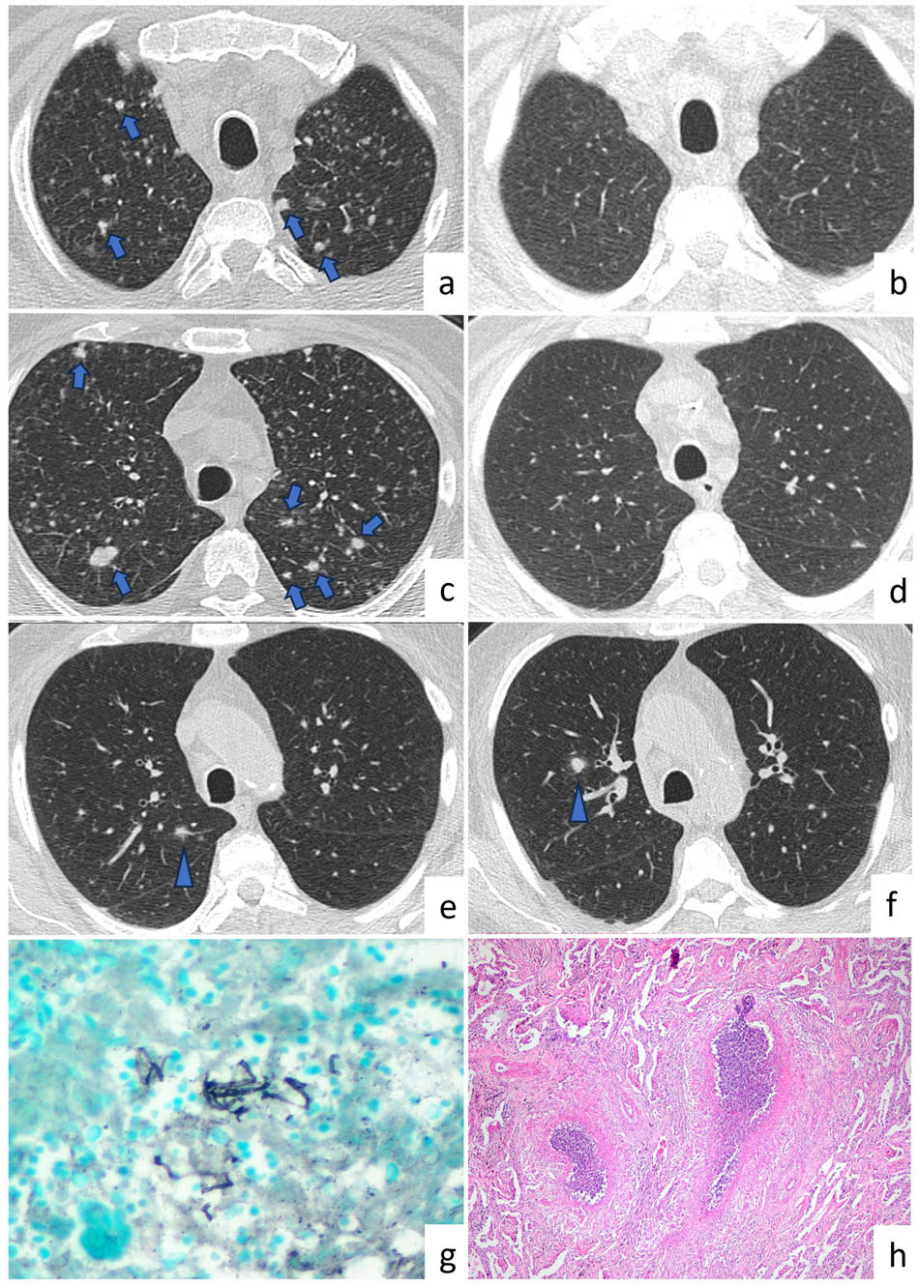
Fungal infection. A 74-year-old patient with a diagnosis of diffuse large B cell lymphoma and fever. Multiple lung nodules located predominantly in upper lobes are seen on CT images (**a**, **c**, **e**, **f**, arrows). After 1 month of voriconazole treatment, the nodules disappeared on follow-up CT images (**b**, **d**). Halo sign is observed in nodules in the right upper lung lobe as peripheral ground-glass opacity (**e**, **f**, arrowheads). Photomicrograph of a specimen from the patient shows *Aspergillus* species fungal hyphae by GMS histochemical stain (Gomori methenamine silver (GMS) × 200) (**g**). Fungal infection can form necrotizing granulomas (Hematoxylin and eosin (H&E) × 40) (**h**)

The radiological features of fungal infections can vary. Pulmonary involvement mostly presents as a single lung nodule in immunocompetent patients, although consolidation and multiple pulmonary nodules are also observed. Although fungal infection can affect any lobe of the lung, the upper lobes are the most common sites. The nodules may be solid or subsolid (Fig. 2). Calcification is not usually present [6]. Cavity, vacuole sign, air crescent sign, halo sign (Fig. 2), satellite lesions, and air bronchograms have been commonly reported in pulmonary nodules associated with fungal infection [7].

Distinguishing pulmonary fungal infection from metastasis in patients with known malignancy is challenging, especially when the fungal infection is accompanied by multiple pulmonary nodules. Hilar and mediastinal lymphadenopathy may accompany fungal infection. Metastases are usually located in the lower lobes, whereas fungal nodules are commonly located in the upper lobes. The ground glass opacity surrounding the nodule, known as the halo sign, is not specific for fungal infection and is observed in hemorrhagic lung metastases [7]. Multiple pulmonary nodules predominantly located in the upper lobes, along with a halo sign in patients with neutropenic fever, suggest fungal infection instead of metastatic lung disease. Additionally, laboratory tests, bronchoalveolar lavage (BAL) culture, and good response to antifungal therapy can support fungal infection diagnosis.

### Pulmonary hydatid cyst (PHC)

Hydatid disease, which is a parasitic infection (zoonosis) caused by *Echinococcus granulosus*, is characterized by cystic lesions. These lesions are common in the liver and lungs but can rarely occur in other parts of the body. Lung parenchyma involvement is frequent in children and young adults. In adults, the lung is the second most commonly affected site after the liver [8, 9]. Most patients with PHC are asymptomatic. Uncomplicated PHCs are most commonly diagnosed incidentally on imaging. In symptomatic cases, the symptoms may vary depending on the organ involved and the size of PHC and include cough, chest pain, and hemoptysis. Cases of ruptured hydatid cysts may present with systemic symptoms, such as fever, fatigue, and weight loss [10]. Diagnosis is based on clinical and radiological findings, as well as serological examination.

**Fig. 3:**
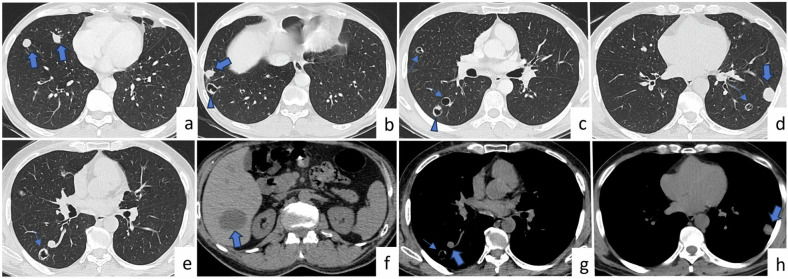
Pulmonary hydatid cyst. A 58-year-old patient with no known disease is admitted to the hospital with cough, fever, and malaise. Multiple nodules are seen at different locations on axial (**a**–**e**) chest CT images. These nodules have variable sizes and shapes. On axial chest CT images (mediastinal window) (**g**, **h**), these nodules are seen as low density (thick arrows). There is also a hypodense lesion in the liver on abdominal CT images (**f**, thick arrow). Some of the lesions are completely ruptured, and some of them represent contained rupture (**b**–**e**, thin arrows and arrowheads). Mass within cavity sign (**b**, **c**, arrowheads), and empty cyst sign (**c**–**e**, **g**, thin arrows) are complete rupture signs of pulmonary HC. Echinococcus IgG was positive

Plain chest radiography, ultrasonography, CT, and magnetic resonance imaging can be used for the diagnosis of PHC. PHCs are usually seen in the lower lobes (55–70%) and can be multiple (30%) or bilateral (20%). These nodules may appear in different forms depending on whether they are complicated (contained rupture, complete rupture, or superinfection) or uncomplicated [8, 11, 12]. Uncomplicated PHCs are seen as homogeneous, well-defined, round, or oval nodules or masses (Fig. 3) and are characterized by low-density Hounsfield unit measurements consistent with fluid content. Complicated cysts may exhibit a meniscus sign, mass within cavity sign, empty cyst sign, air crescent sign, combo or onion peel sign, and water-lily sign [8, 9, 13, 14] (Fig. 3). The contrast-enhanced CT of the chest may show a thin enhancing rim of the cyst wall.

Multiple pulmonary nodules are rare in patients with PHC but are observed in patients with a history of malignancy. In addition to the low density of the existing nodules, the previously mentioned specific signs showing the collapsed endocyst and floating membranes and the presence of daughter cysts within the PHCs are crucial for distinguishing pulmonary metastases. Additionally, the presence of a cystic lesion in the liver within the chest CT scans and serological tests may help in the differential diagnosis. In contrast to other cases, biopsy should be avoided in these cases due to the risk of rupture and contamination [15].

## Immune-inflammatory diseases

### Sarcoidosis

Sarcoidosis is a multisystemic granulomatous disease of unknown etiology and is usually seen in patients aged 20–40 years. The disease can affect any system or organs, including the lungs, lymph nodes, skin, eyes, joints, bones, salivary glands, central nervous system, and muscles. Lung involvement is the most common presentation, with a rate of more than 90% [16, 17]. Although the clinical presentation varies according to the involved organ, patients with pulmonary involvement usually present with cough and dyspnea. Sarcoidosis is distinguished by the presence of non-infectious, non-caseating granulomas, primarily consisting of macrophages that transform into epithelioid cells, which then merge to create multinucleated giant cells [18].

**Fig. 4:**
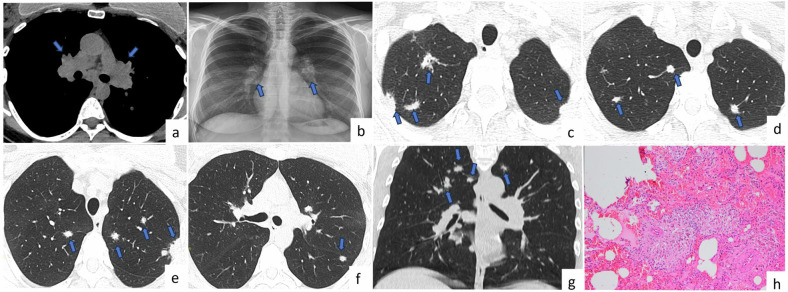
Sarcoidosis. A 45-year-old female patient with a complaint of cough was diagnosed with pulmonary sarcoidosis. Axial chest CT image (mediastinal window) (**a**) shows bilateral hilar (arrows) lymphadenopathies. Posteroanterior chest radiograph (**b**) shows bilateral hilar lymphadenopathy (arrows). Axial (**c**–**f**) and coronal (**g**) CT images show bilateral multiple pulmonary nodules mostly observed in the upper lobes peripherally or centrally (arrows). Fewer nodules are observed in the lower lobes of both lungs. Photomicrograph shows non-necrotizing, well-formed granulomas in the lung parenchyma (H&E × 100) (**h**)

The common radiological findings are hilar and mediastinal lymphadenopathy with or without associated lung parenchymal involvement (Fig. 4). Parenchymal involvement is usually seen as micronodules with perilymphatic distribution in the early stage of the disease, and parenchymal fibrosis in the late stages. However, micronodules may coalesce over time and form macronodules.

Multiple nodular sarcoidosis, which is a rare presentation of pulmonary sarcoidosis with incidence rates varying from 2.4 to 4% [19], usually manifests as multiple, bilateral pulmonary nodules or masses mostly in the perihilar and peripheral region of the lungs [20, 21] (Fig. 4). Additionally, the predominance of nodules in the upper and middle lobes can aid in differential diagnosis [22]. The radiological findings of nodular sarcoidosis are not specific and resemble those of other nodular pulmonary diseases.

Sarcoidosis is known as the “great mimicker” in radiology. Although the presentation of sarcoidosis with multiple pulmonary nodules is rare, it can also mimic metastatic lung lesions. The presence of accompanying mediastinal lymphadenopathy, especially in bilateral hilar and right paratracheal stations, known as the Garland triad, is the most significant radiological finding supporting sarcoidosis diagnosis instead of pulmonary metastatic disease diagnosis [23]. However, in patients with known malignancies, histopathological diagnosis is usually the only way to differentiate metastases from sarcoid nodules.

### Pneumoconiosis

Pneumoconiosis results from the inhalation and accumulation of dust particles in the lungs, leading to tissue reactions. This pathological condition encompasses a diverse range of conditions in terms of their causes, prevalence, and clinical symptoms. Silicosis, coal worker pneumoconiosis, and asbestosis are the most common types of pneumoconiosis [24]. Pneumoconiosis is diagnosed based on the occupational history of exposure to inorganic dust and pathological and radiological evidence. Pathological findings in pneumoconiosis vary depending on the causative dust, with the most common manifestation being fibroinflammation [25].

**Fig. 5:**
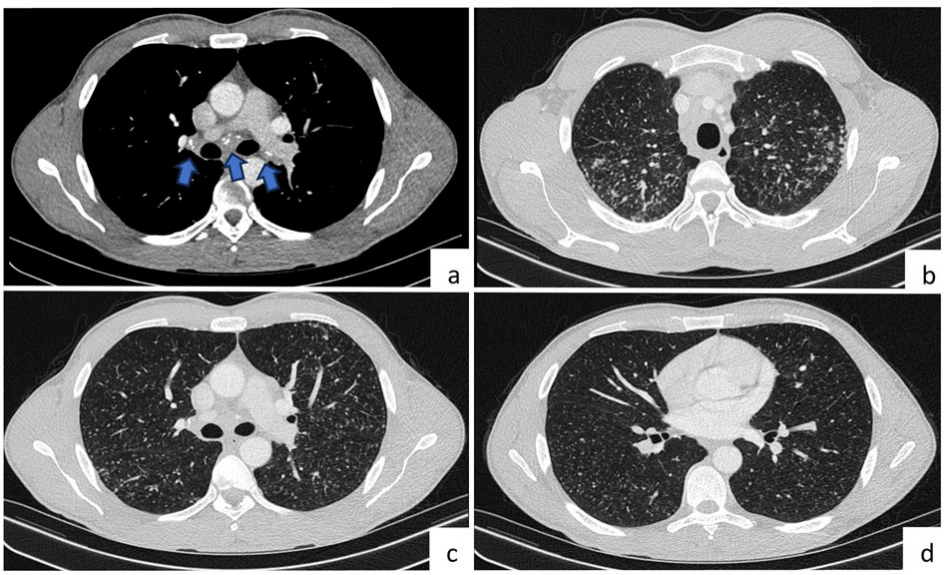
Pneumoconiosis. A 32-year-old male patient, who has no known disease and works as a dental technician, had incidentally detected lung CT findings compatible with silicosis. Axial lung CT images show calcified lymph nodes in the mediastinum and bilateral hilar regions (**a**, arrows), and multiple millimetric solid nodules predominantly in the upper lobes and periphery of both lungs (**b**–**d**)

The radiological features of pneumoconiosis vary widely within the group, often involving similar or distinct patterns of lung involvement among the diseases. Chest CT scans, which can accurately identify these patterns, are important for pneumoconiosis diagnosis. On CT scans, small rounded or irregular opacities, ground glass opacities, reticulation, pleural thickening, hilar and mediastinal lymph node enlargement, progressive massive fibrosis, conglomerate masses, thickened intralobular and interlobular lines, subpleural curvilinear lines, small cystic spaces, honeycomb pattern, and bronchial wall thickening may be observed [26, 27]. Silicosis, coal worker pneumoconiosis, berylliosis, talcosis, and siderosis typically present with diffuse small parenchymal lung nodules. Although the radiological features of diseases in this group may share similarities, characteristic distinctions can be crucial for differentiation. In individuals with silicosis, predominant radiological findings comprise numerous well-defined small nodules with a predilection for the upper lobe and posterior regions. On CT scans, these nodules are commonly located in centrilobular, paraseptal, and subpleural regions, exhibiting a perilymphatic distribution pattern. Before the manifestation of nodular lesions in lung parenchyma, enlargement of hilar and mediastinal lymph nodes can occur. The presence of calcification in lymph nodes, especially in a characteristic eggshell configuration, indicates the diagnosis of silicosis [26, 28] (Fig. 5). The radiological findings of coal worker pneumoconiosis are similar to those of silicosis. In particular, coal worker pneumoconiosis is characterized by small multiple nodules. However, these nodules in coal worker pneumoconiosis often have less sharply defined margins and exhibit a granular appearance when compared with those observed in silicosis [29]. Berylliosis, which is a chronic granulomatous lung disorder caused by exposure to beryllium particles, is characterized by reticulonodular opacities primarily affecting the middle and upper lung regions accompanied by honeycomb pattern or mass lesions. CT findings in berylliosis may resemble those observed in other granulomatous lung conditions, such as sarcoidosis [30]. Talcosis manifests as diffuse small nodules and perihilar conglomerate masses, while siderosis is characterized by small nodules, particularly prominent in the middle third of the lungs and perihilar regions [31, 32].

Distinguishing this group of diseases from metastases can be challenging in patients with multiple pulmonary nodules, especially in those with primary malignancies. Differential diagnosis of pneumoconiosis can be achieved using clinical and pathological findings, characteristic radiological features, occupational information, and information on recent inorganic dust inhalation.

### Rheumatoid pulmonary nodules

Rheumatoid arthritis (RA) can be associated with pulmonary involvement through different mechanisms. Pleural involvement, rheumatoid nodules, and diffuse interstitial involvement are common manifestations of RA. Additionally, pulmonary hypertension and airway diseases, such as bronchiolitis obliterans and follicular bronchiolitis, are observed in patients with RA patients [33]. Rheumatoid nodules can be associated with cavitation and are called necrobiotic pulmonary nodules. Rheumatoid pulmonary nodules are rare and are associated with a good prognosis. Approximately 70% of patients with RA in the Brigham RA Sequential Study registry are reported to be seropositive, which is defined by the presence of either rheumatoid factor (RF) or anti-citrullinated protein antibodies (ACPA) [34]. The probability of the occurrence of subcutaneous nodules and seropositivity is high in patients with RA exhibiting lung nodules. The age of these patients is generally lower than that of patients with a history of cancer. Additionally, the frequency of rheumatoid pulmonary nodules is high in male patients and smokers [35]. The characteristic histopathological features of rheumatoid lung nodules involve a central region of fibrinoid necrosis surrounded by arranged epithelioid cells, which are enveloped by a border comprising lymphocytes, plasma cells, and fibroblasts [36].

**Fig. 6:**
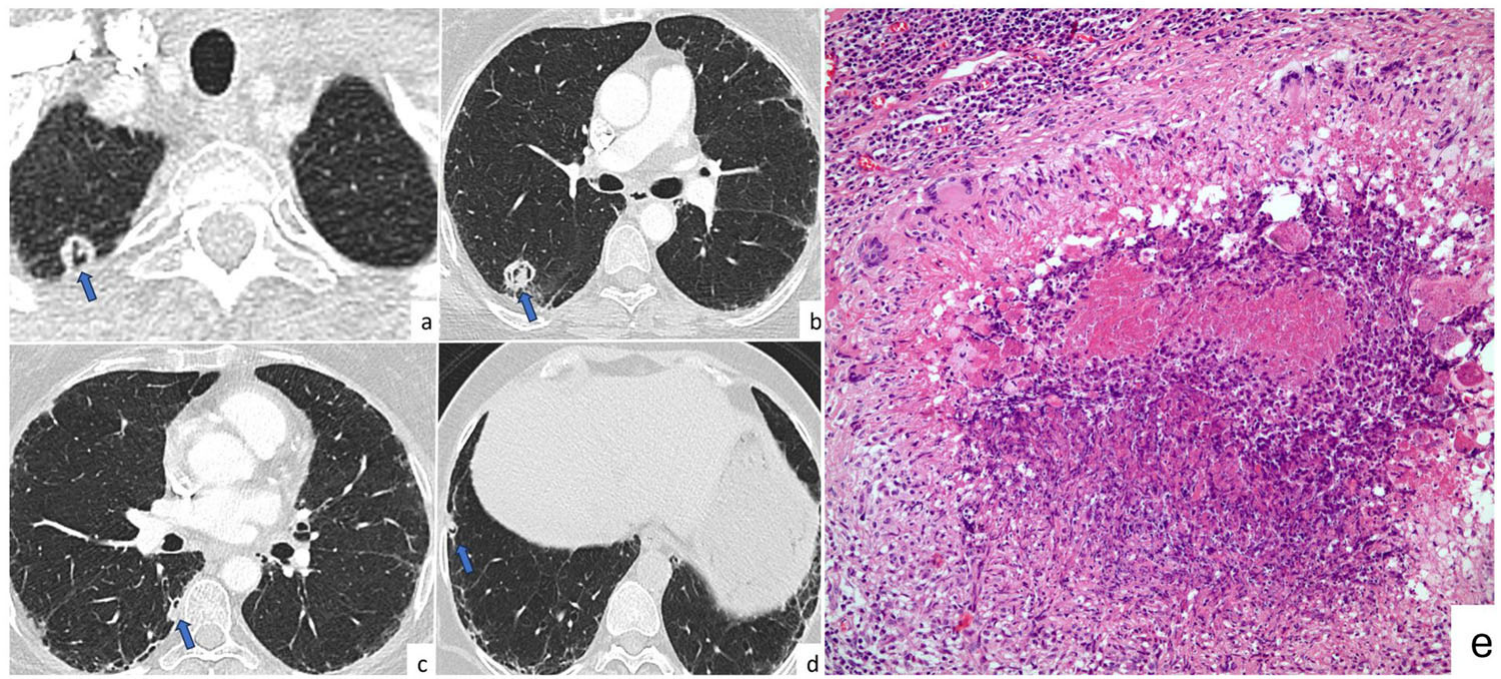
Rheumatoid pulmonary nodules. A 66-year-old female with a history of rheumatoid arthritis and no malignancy, axial CT images (**a**–**d**); show multiple, cavitary, peripheral, and subpleural nodules compatible with rheumatoid nodules (arrows). In addition, due to the interstitial involvement of the disease, diffuse, peripheral reticular densities are observed in both lungs. Photomicrograph shows fibrinoid and suppurative necrosis is surrounded by epithelioid histiocytes and multinucleated giant cells, and it is enveloped by a border consisting of fibroblasts and lymphoplasmacytes (H&E × 100) (**e**)

A review of the radiological findings revealed that the size of rheumatoid nodules ranges from millimeters to several centimeters and that these nodules are single or multiple, solid or cavitary (approximately 50%), and generally located in middle and upper zones and peripheral and subpleural locations [37] (Fig. 6). Low or medium FDG uptake on PET-CT scan is usually observed in RA nodules [35]. Thus, metastases must be excluded from differential diagnosis of multiple nodules in patients with a history of malignancy and RA. This is because patients with RA nodules are asymptomatic and do not require treatment unless infection or bronchopleural fistula develops. Differential diagnosis of rheumatoid pulmonary nodules is simple in cases of typical radiological findings. However, a biopsy may be needed in some rare cases. The location of nodules and follow-up images may help differentiate RA nodules from metastatic nodules.

### Granulomatosis with polyangiitis (GPA)

GPA, previously known as Wegener’s granulomatosis, is a multisystemic necrotizing non-caseating granulomatous vasculitis. This disease mostly affects small to medium-sized arteries, capillaries, and veins. GPA usually occurs in middle-aged patients but can occur in any age group. GPA can involve any organ, the most common of which is the upper respiratory tract. The lungs and kidneys are the other frequently involved organs [38]. In pathological specimens, GPA may present with necrosis, granulomatous inflammation, and vasculitis individually, although these findings may not always coexist [39].

**Fig. 7:**
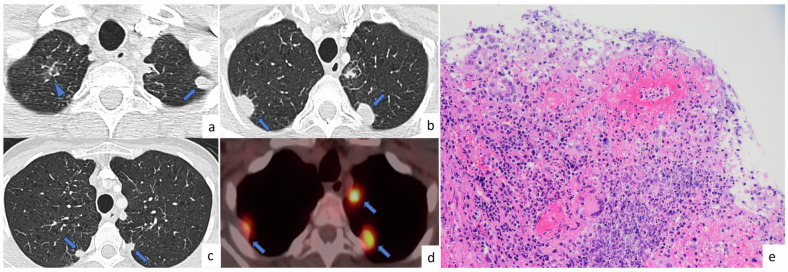
Granulomatosis with polyangiitis. On axial CT images (**a**–**c**) obtained at various levels, there are bilateral multiple lung nodules (arrows), and ground glass opacity located in right upper lobe (**a**, arrowhead). There is high FDG uptake on PET-CT image (**d**, arrows). Nodules may be located centrally or peripherally. Photomicrograph shows vasculitis with fibrinoid necrosis, dirty necrosis, and scattered multinucleated giant cells (H&E × 100) (**e**)

Lung manifestations can include multiple nodules, pulmonary hemorrhage, reticulonodular pattern, and peripheral wedge-like consolidation. Pleural effusions are seen in 10–25% of cases. GPA most commonly presents with multiple and bilateral pulmonary nodules, occurring in approximately 40–70% of cases. These nodules are not location-specific. The size of the nodules ranges from 1 mm to 10 cm but is generally in the range of 2–4 cm. Cavitation can be seen, especially in nodules larger than 2 cm. The cavity wall may be thin or thick [40] (Fig. 7). Halo sign and reverse halo sign may accompany the nodules.

GPA nodules can be misdiagnosed as metastases, especially in patients with a history of malignancy. The serum titers of c-antineutrophil antibodies against protease 3 in cytoplasmic granules (c-ANCA) are upregulated in patients with GPA and can be used to differentiate the nodules from metastases and assess disease activity [40]. The presence of cavitation in larger nodules may suggest GPA, although it is also observed in some metastases, especially in those originating from the gastrointestinal system and squamous cell carcinoma [41].

### Cryptogenic organizing pneumonia (COP)

Organizing pneumonia (OP), which is a histological pattern of alveolar inflammation, can be classified as primary and secondary OP according to etiology. The primary (idiopathic) form is called COP. The etiological factor for COP is unknown. Meanwhile, secondary OP has several etiological factors, such as infections, connective tissue diseases, malignancy, chemotherapy, radiotherapy, organ transplantation, inhalation of harmful gases, and certain professions [42, 43]. The diagnosis of COP is delayed as patients exhibit no specific symptoms. Patients may present with flu-like symptoms (fever, cough, fatigue) or shortness of breath. COP is not related to smoking. The precise pathogenic mechanisms of COP have not been elucidated. However, alveolar epithelial damage triggered by an unidentified stimulus is hypothesized to promote the leakage of plasma proteins into the alveolar space, recruiting inflammatory cells [44].

Although imaging is important in the diagnosis of COP, it may reveal different patterns. The main radiological finding of COP is patchy air-space consolidation, which may show a migratory nature. However, this consolidation can have ground glass opacities with or without reverse halo (atoll) sign, masses, or nodules. Bronchial wall thickening, bronchial dilatation, mediastinal lymphadenopathy, and pleural effusion may also be observed [45, 46].

**Fig. 8:**
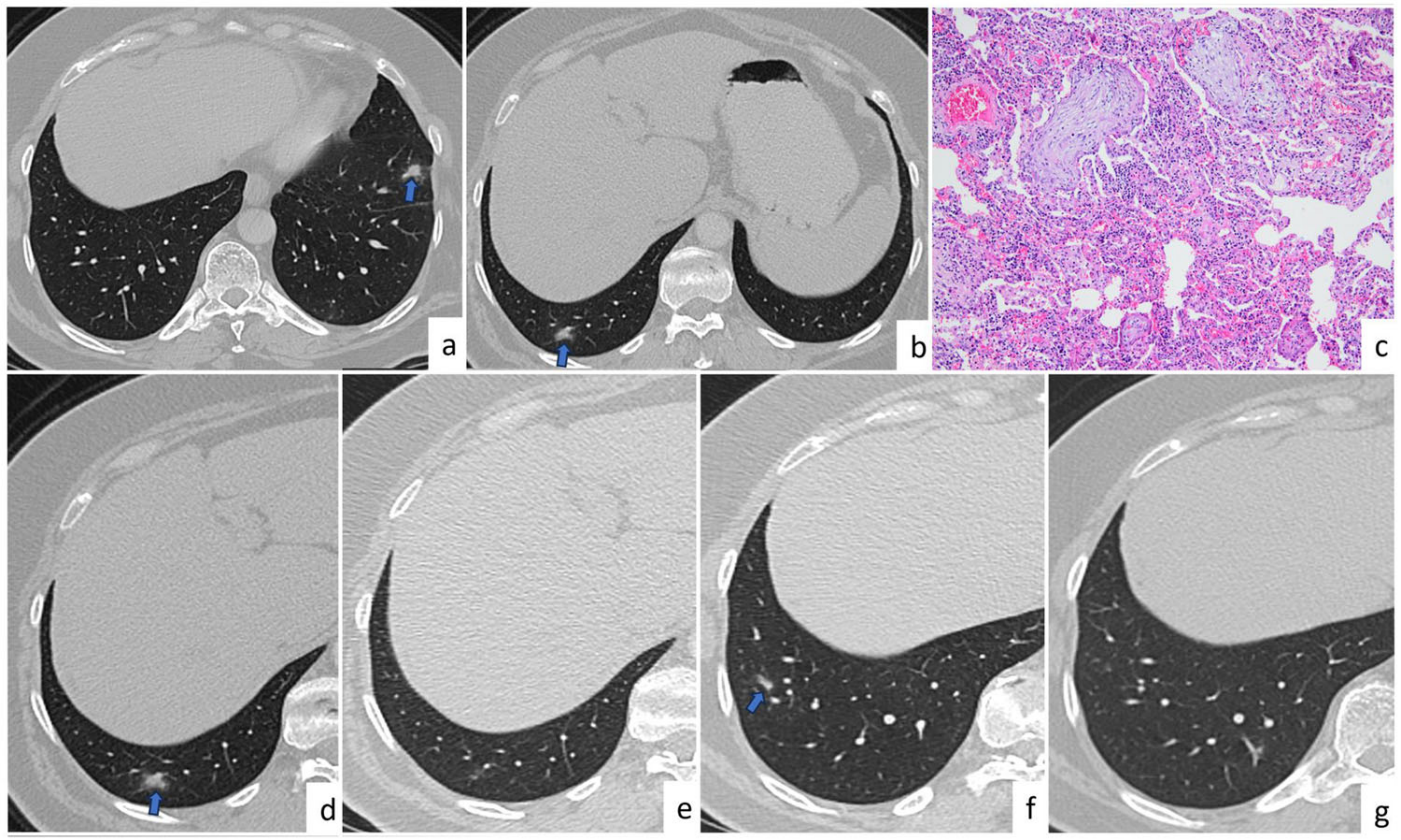
Cryptogenic organizing pneumonia. A 57-year-old patient who was previously diagnosed with rectal cancer and underwent lung metastasectomy. The follow-up lung CT revealed new poorly defined nodules in the lower lobes of both lungs (**a**, **b**) (arrows). Photomicrograph shows fibroblastic foci protruding into alveoli. Lymphocytic inflammation in the interstitial area and foamy macrophages in the alveoli are seen (H&E × 100) (**c**). On axial CT images (**d**–**g**) of the same patient obtained at different time points, the nodules observed in the right lower lobe (**d**, **f**, arrows) disappeared 2 months later (**e**, **g**)

Solitary or multiple nodules and mass-like presentation are seen in the atypical pattern of COP. A nodular pattern is seen in 15–50% of patients [46]. Although the cause is not clear, the nodular pattern is common in immunocompromised patients when compared with that in immunocompetent patients. The size of the lesions can vary from micronodules to masses. Nodules can be solid, partially solid, or ground glass and have well-defined, poorly defined, or even spiculated borders with no specific distribution [47] (Fig. 8).

When OP presents as a nodule, malignancy must be excluded as an etiological factor, especially in cases of large and spiculated nodules. Additionally, differentiating the nodular form of OP from metastases can be challenging as it can appear as multiple nodules, especially in patients with primary malignancy and a history of radiotherapy or chemotherapy that may cause OP. In these cases, differential diagnosis can be achieved through follow-up imaging and biopsy.

### Pulmonary Langerhans cell histiocytosis (PLCH)

PLCH is an isolated form of Langerhans cell histiocytosis with granulomatous infiltration in the distal bronchial walls of the lung. Additionally, PLCH is a rare form of interstitial lung disease (less than 5%) with unknown etiology. PLCH can occur at any age but is mostly observed in young adults who smoke (90–95%). Patients are usually symptomatic with no productive cough, dyspnea, or fever. Spontaneous pneumothorax may be the initial manifestation in 10–20% of patients with PLCH [48, 49]. PLCH is associated with variable prognosis and generally achieves remission or stabilization. However, the disease progresses in a small group of patients (15–25%) [50, 51]. PLCH is diagnosed based on typical radiological findings, especially in young patients with a smoking history. Biopsy may be required in a considerable number of cases. The typical pathology observed in PLCH involves the clustering of Langerhans cells and diverse inflammatory cells within the small airways, resulting in the development of nodular inflammatory lesions. Additionally, advanced stages are associated with cystic lung destruction and scarring of airways [52].

**Fig. 9:**
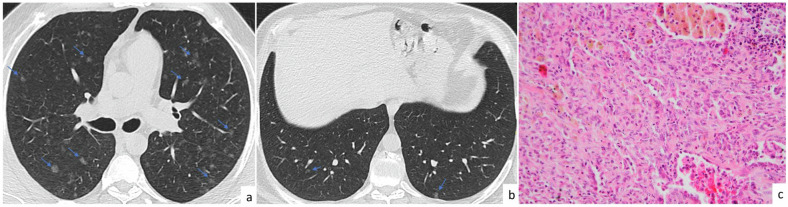
Pulmonary Langerhans cell histiocytosis. Axial CT images of a 38-year-old male smoker with no known malignancy show multiple micronodules (arrows) predominantly in bilateral upper lobes due to PLCH (**a**, **b**). Photomicrograph shows the clustering of Langerhans cells within the small airways, resulting in the development of nodular inflammatory lesions (H&E × 200) (**c**)

Imaging has a critical role in the diagnosis of PLCH. PLCH appears as multiple bronchiolocentric lesions at the upper-middle lung section, usually sparing both lung bases and costophrenic angles. Pulmonary involvement can be observed as multiple nodules, micronodules, cystic lesions, and rarely as alveolar consolidation or a single nodule. The involvement varies depending on the disease phase. In the early period (inflammatory phase), PLCH usually presents as bilateral, bronchiolocentric, symmetrical, upper lobe-predominant nodules with a size in the range of 1–10 mm (Fig. 9). The nodules usually have irregular and different shapes with their surrounding regions associated with a ground glass opacity. As the inflammatory activity decreases, these nodules become thick-walled (> 2 mm) and subsequently thin-walled (< 2 mm) cysts. These cavitary lesions and cysts can have different shapes, such as round, irregular, bilobed, and branched. The final stage is characterized by fibrocystic disease with predominantly upper and middle lobe involvement [51].

PLCH nodules can be observed during serial imaging in patients with cancer. Thus, the differential diagnosis of PLCH, especially the nodular form, is challenging. The key radiological feature differentiating PLCH from pulmonary metastases is the distribution of the nodules. The predilection of upper and mid zones with regional sparing of the costophrenic angles suggests PLCH instead of metastases that predominantly involve the lower zones. The development of cavitation and cysts on follow-up images and smoking history can also help in differential diagnosis. However, cavitary metastases in some malignant diseases or the development of cavitation in some metastatic pulmonary nodules after chemotherapy should also be considered. A biopsy may be required when the distinction is challenging in patients with a history of malignancy.

## Neoplastic diseases

### Nodular pulmonary amyloidosis

Amyloidosis is characterized by the accumulation of fibrils of amyloid, an extracellular protein, in many organs. The disease can be primary or secondary. Primary pulmonary amyloidosis is confined to the lungs. Pulmonary amyloidosis may be part of a systemic process (80–90%) or localized only to the lung (10–20%) [53]. The samples of pulmonary amyloidosis are characterized by aberrant accumulation of amyloid fibrils in the extracellular space of the lung tissue [54].

**Fig. 10:**
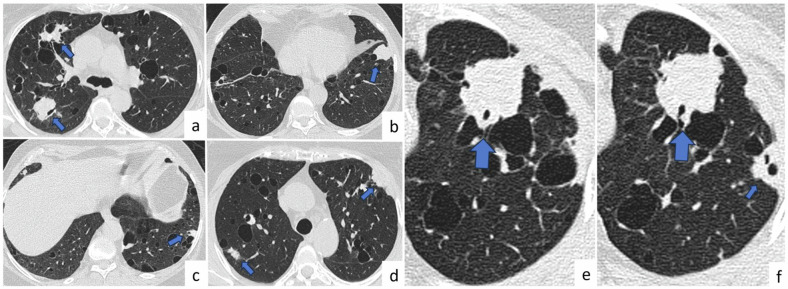
Nodular pulmonary amyloidosis. A 49-year-old female patient with known diagnoses of Sjogren’s syndrome and lymphoid interstitial lung disease. Axial CT images (**a**–**f**); show multiple pulmonary nodules in variable sizes and shapes (arrows). There is no significant progression in size of the mass located in the left upper lobe between 2004 (**e**) and 2008 (**f**). The diagnosis of amyloidosis was confirmed with CT-guided biopsy

Lung involvement may manifest as diffuse reticulonodular interstitial thickening, consolidations, or solitary and multiple parenchymal nodules. Nodular parenchymal involvement is more common than diffuse parenchymal involvement. These nodules may be calcified or cavitary and may increase in size over time [55, 56]. Nodular opacities are typically present in the lower lobes at the periphery and may have sharp, smooth, and lobulated contours. Central or irregular calcification may be observed in approximately 50% of the nodules. These nodules exhibit size in the range of 0.5–15 cm. Slow growth of nodules may be observed, but no regression is expected [57] (Fig. 10). Mediastinal lymphadenopathy may sometimes be observed. In systemic amyloidosis, the combination of lymphadenopathy and parenchymal involvement is common [58]. Pulmonary nodular amyloidosis can mimic metastases, especially in cases with primary malignancy. However, the presence of cysts or Sjogren’s syndrome [59] and multiple pulmonary nodules indicate amyloidosis rather than metastases. A follow-up CT imaging is suggested for differential diagnosis.

### Pulmonary epithelioid hemangioendothelioma (PEH)

PEH is a rare disease of vascular origin that can affect multiple organs, such as the bone, liver, soft tissue, or lung. In addition to lung involvement, pleural origin has been less frequently described [60]. PEH, which typically affects women, is often asymptomatic and is incidentally discovered. The prognosis of PEH is good despite extensive parenchymal involvement. The histological characteristics of PEH include epithelioid cells with abundant eosinophilic cytoplasm within a fibromyxoid stroma; some of these epithelioid cells may contain intracytoplasmic vacuoles [61].

**Fig. 11:**
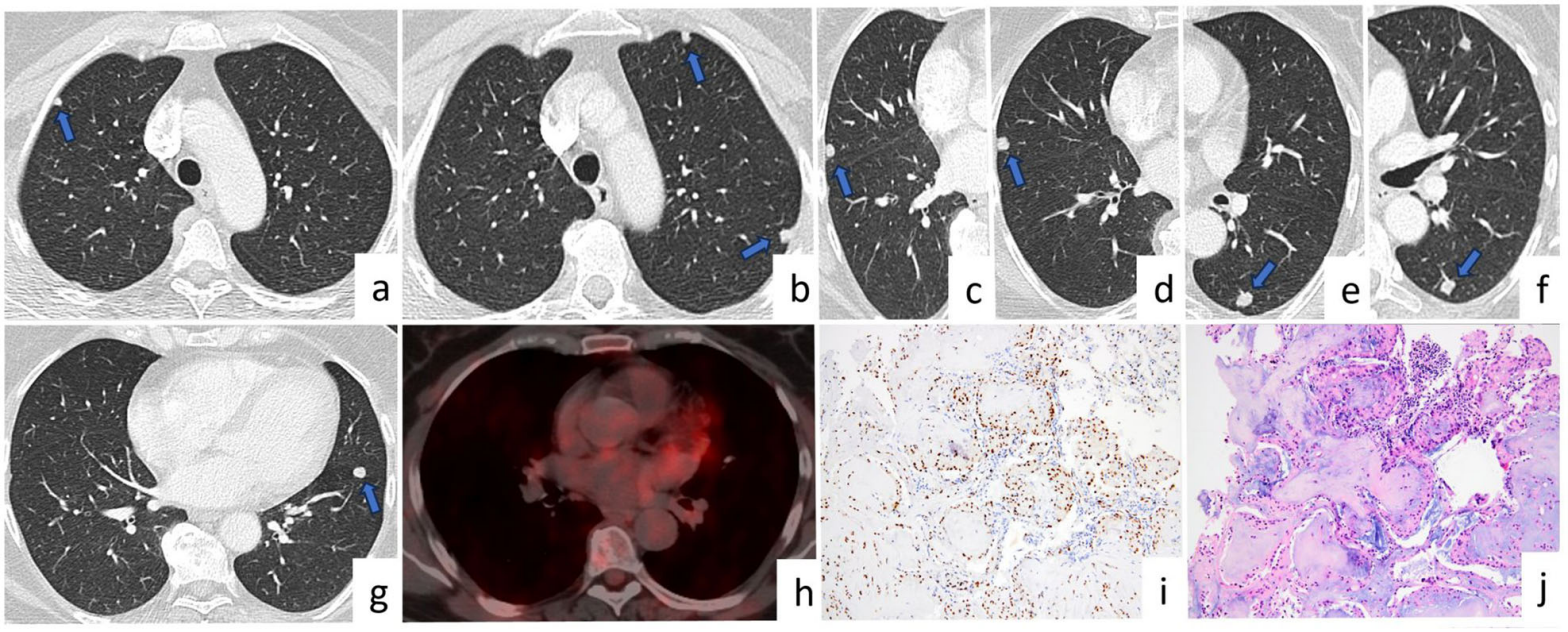
Pulmonary epithelioid hemangioendothelioma. A 61-year-old asymptomatic female patient with incidentally detected multiple lung nodules, without a history of malignancy. Axial CT images (**a**, **b**, **g**), show randomly distributed (central or periphery), well-defined solid nodules in both lungs (arrows). There was no FDG uptake on PET-CT (**h**). On axial CT images (**c**, **d**, **e**, **f**) obtained in 2018 (**c**, **e**) and 2022 (**d**, **f**), there is no significant progression in the size of the nodule located in the right upper lobe (**c**, **d**) and left lower lobe (**e**, **f**). Photomicrograph shows cords of epitheliod cells within a myxoid and hyaline stroma. Tumor cells have uniform round to oval nuclei and abundant eosinophilic cytoplasm (H&E × 100) (**j**). Immunohistochemically, tumor cells express ERG as an endothelial marker (× 100) (**i**)

PEH presents as bilateral randomly distributed, well or ill-defined multiple parenchymal nodules. The nodules are usually located near medium-sized vessels and bronchi. Additionally, calcifications are occasionally observed within the nodules [60]. The size of the nodules is often less than 1 cm but can reach up to 2 cm (Fig. 11). The nodules can exhibit an increased size and FDG uptake on PET-CT scans. Histopathological analysis is required for the diagnosis of PEH as it is rare and associated with ambiguous radiological findings [62].

### Minute pulmonary meningothelial-like nodules (MPMNs)

MPMNs are rare interstitial pulmonary nodules that can be solitary or diffuse. Additionally, MPMNs are benign and are often incidentally discovered. Previously, MPMNs were called pulmonary chemodectomas. However, further studies revealed that MPMNs resemble meningothelial cells and do not contain endocrine granules [63]. MPMNs are acquired and are commonly seen in women aged more than 50 years. MPMNs are characterized by the widespread proliferation of meningothelial cells within the lung tissue [64].

**Fig. 12:**
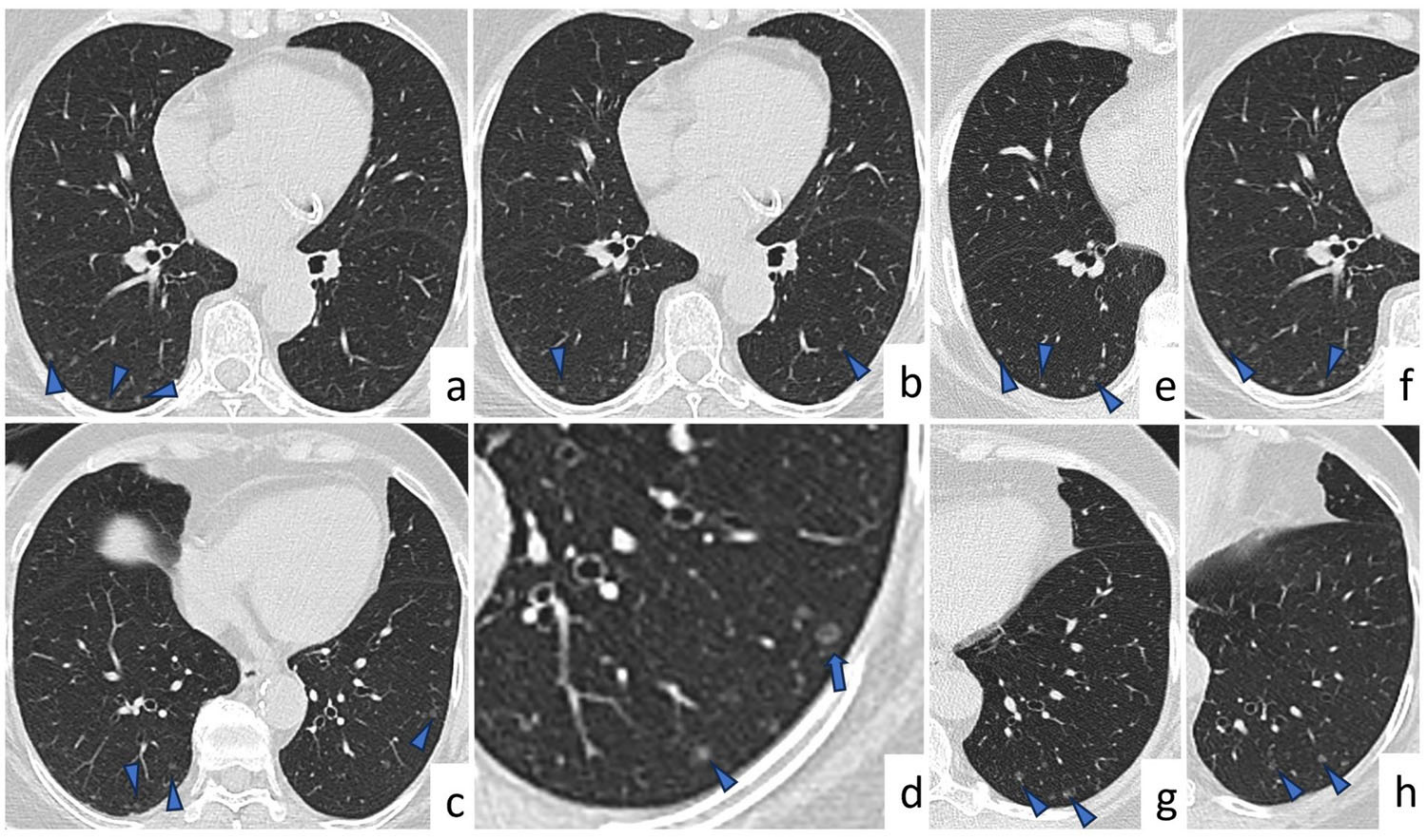
Minute pulmonary meningothelial-like nodules. A 61-year-old female patient suffering from cough and no known history of smoking or malignancy. Axial CT images (**a**–**d**) show small, randomly distributed, low-attenuation multiple nodules (arrowheads). Axial CT image of the left lower lobe (**d**) shows a ring-like nodule with central lucency (arrow) (Cheerio sign). On axial CT images (**e**–**h**) in 2007 (**e**, **g**) and 2022 (**f**, **h**), there is no significant progression in the size and shape of multiple tiny nodules (arrowheads) located in the right lower lobe (**e**, **f**) and left lower lobe (**g**, **h**)

On CT images, MPMNs appear as small, randomly distributed, solid or subsolid multiple nodules, or as tiny rings with central lung attenuation, which is defined as a cheerio sign (Fig. 12). MPMNs usually follow a benign, slow progression but may mimic adenocarcinoma in situ or metastasis. Stability or slow increase in size and number of the nodules and the presence of the cheerio sign may radiologically suggest MPMNs. However, the cheerio sign is not specific for MPMNs and is observed in PLCH and some pulmonary metastases. Therefore, tissue sampling is usually required to establish a definitive diagnosis.

### Diffuse idiopathic pulmonary neuroendocrine cell hyperplasia (DIPNECH)/pulmonary tumorlets

DIPNECH refers to a type of neuroendocrine cell proliferation in the lung. DIPNECH, carcinoid tumorlets, and typical carcinoids are included in the neuroendocrine tumor subgroup and are distinguished based on their distinct morphological, immunochemical, and molecular features [65]. Carcinoid tumorlets are distinguished from DIPNECH based on the extension beyond the basement membrane of the epithelia and have a size of ≤ 5 mm. The relationship between tumorlet and carcinoid is not clear. Carcinoids may develop from tumorlets [66]. Tumorlets usually occur in middle-aged women and have a good prognosis. Although Tumorlets are usually asymptomatic, they are accompanied by dyspnea and chronic cough. Histological analysis revealed that DIPNECH can appear as a widespread proliferation of dispersed neuroendocrine cells forming small nodular clusters or as a linear proliferation of neuroendocrine cells [67].

Characteristic CT features of DIPNECH are multiple bronchocentric, well-circumscribed solid nodules predominantly involving lower zones with lobular or regional air trapping. Mucous plugging, bronchiectasis, atelectasis, and nodular bronchial wall thickening are also observed. Air trapping is useful for distinguishing DIPNECH from pulmonary metastases [68] (Fig. S1). Furthermore, the high sensitivity of gallium-dodecane tetraacetic acid-peptide (Ga-DOTA-Peptide) PET-CT may aid in identifying accompanying larger nodules representing carcinoid tumors [69].

### Pulmonary sclerosing pneumocytoma (PSP)

PSP is a rare benign tumor and is also known as pulmonary sclerosing hemangioma. The etiology and pathogenesis of PSP are unclear. Most patients with PSP are asymptomatic. PSP is frequent in women aged 30–50 years. The histopathological analysis of PSP typically reveals two distinct cell types (cuboidal surface cells and round stromal cells). Four different histological growth patterns have been described that can coexist (papillary, sclerotic, solid, and hemorrhagic) [70, 71].

Generally, PSP presents as a well-defined, round, or oval, juxta-pleural nodule with strong, homogeneous enhancement [72]. Additionally, the nodules may exhibit crescentic radiolucency at the periphery (air-meniscus sign) and a marginal pseudocapsule sign resulting from compression of adjacent parenchyma [70]. Although pulmonary sclerosing hemangiomas are rare, most of them appear as solitary pulmonary nodules. The presentation of bilateral multifocal nodules is extremely rare, accounting for only 4% of cases. PSP nodules may slowly grow in size with low or intermediate FDG uptake on PET-CT scans, although FDG-avid PSP is reported in exceptional cases [73–76] (Fig. S2). The differential diagnosis of PSP is challenging in patients with a known history of malignancy. Thus, pathological diagnosis is the gold-standard method for the accurate diagnosis of this rare disease.

## Conclusion

A wide range of infectious, immune-inflammatory, and neoplastic diseases may present with multiple pulmonary nodules and mimic metastatic disease presentations. In patients with primary malignancies, distinguishing between non-metastatic and metastatic pulmonary nodules requires evaluating clinical and laboratory findings, nodule characteristics (such as size, location, margins, composition, and growth rate), and additional pulmonary and extrathoracic findings for early diagnosis and effective treatment.

## Supplementary information

ELECTRONIC SUPPLEMENTARY MATERIAL

## Abbreviations

ACPA: Anti-citrullinated protein antibodies
BAL: Bronchoalveolar lavage
C-ANCA: Serum c-antineutrophil antibodies against protease 3 in cytoplasmic granules
COP: Cryptogenic organizing pneumonia
CT: Computed tomography
DIPNECH: Diffuse idiopathic pulmonary neuroendocrine cell hyperplasia
FDG: Fluorodeoxyglucose
GPA: Granulomatosis with polyangiitis
MPMN: Minute pulmonary meningothelial-like nodules
OP: Organizing pneumonia
PEH: Pulmonary epithelioid hemangioendothelioma
PET-CT: Positron emission tomography-computed tomography
PHC: Pulmonary hydatid cyst
PLCH: Pulmonary Langerhans cell histiocytosis
PSP: Pulmonary sclerosing pneumocytoma
RA: Rheumatoid arthritis
RF: Rheumatoid factor
TB: Tuberculosis
