# Supplementary material for: Non-metastatic causes of multiple pulmonary nodules
Source: Insights Imaging. 2024 Nov 29;15:288. doi: 10.1186/s13244-024-01856-9 (PMC11607223; doi:10.1186/s13244-024-01856-9)
Supplement: Supplementary file 1 — ELECTRONIC SUPPLEMENTARY MATERIAL [file 13244_2024_1856_MOESM1_ESM.pdf]

Non-metastatic causes of multiple pulmonary nodules

ELECTRONIC SUPPLEMENTARY MATERIAL

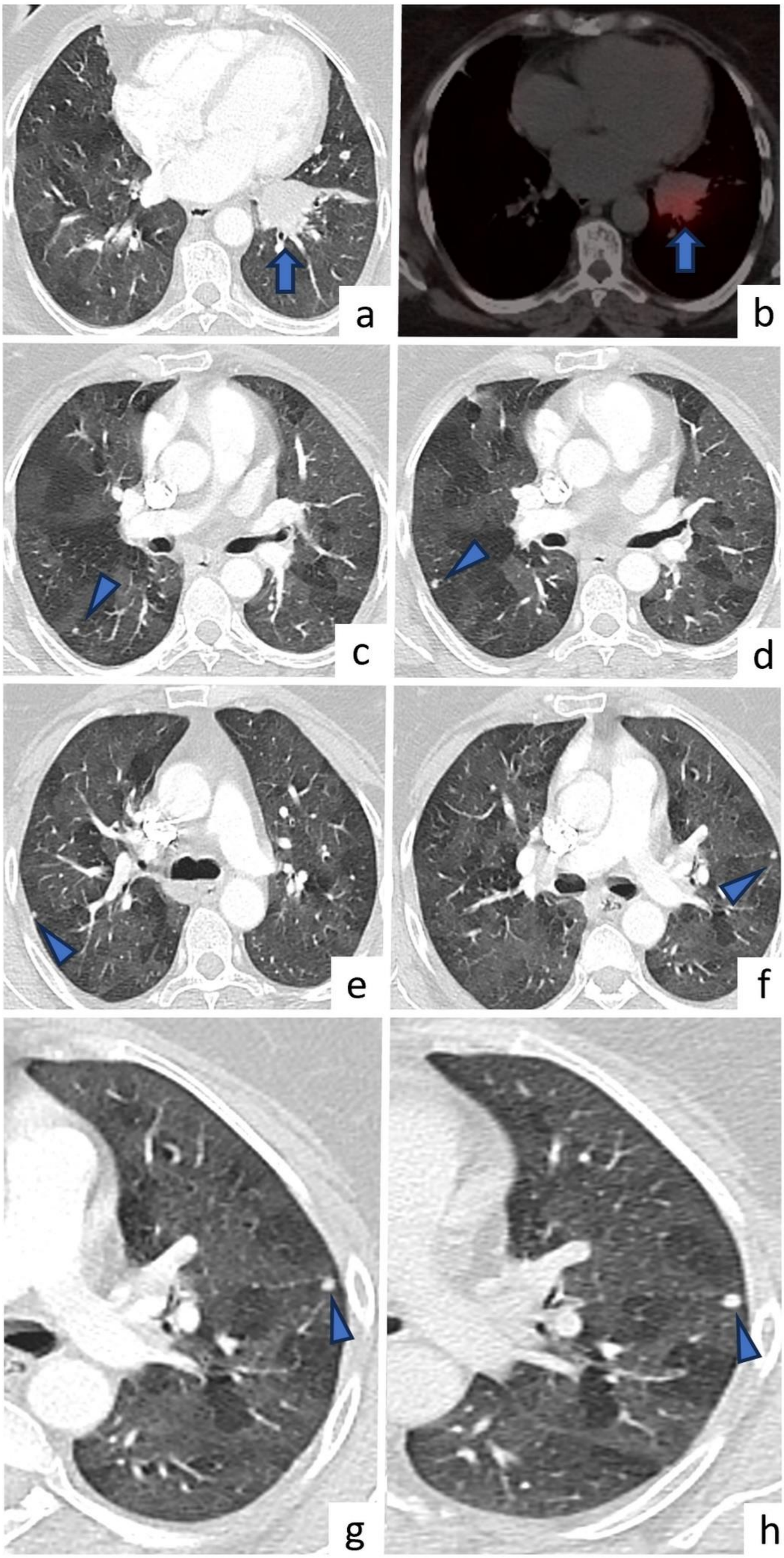

Fig. S1. Diffuse idiopathic pulmonary neuroendocrine cell hyperplasia. A 49-year-old female patient with diarrhea and facial flushing. On axial CT image **(a)** there is a centrally located biopsy-confirmed carcinoid lesion in the left lower lobe (**arrow**). There is a low uptake on Ga-68 DOTATATE PET-CT (SUVmax: 2.8) **(b).** **Axial** CT images in lung window **(c-f),** show multiple and randomly distributed solid nodules compatible with pulmonary tumorlets (**arrowheads**). In addition, a mosaic attenuation pattern due to air trapping is also seen. On axial CT images **(g, h)** obtained in 2018 **(g)** and 2022 **(h)**, there is no significant progression in the size of the nodule located in the left upper lobe (**arrowheads**).

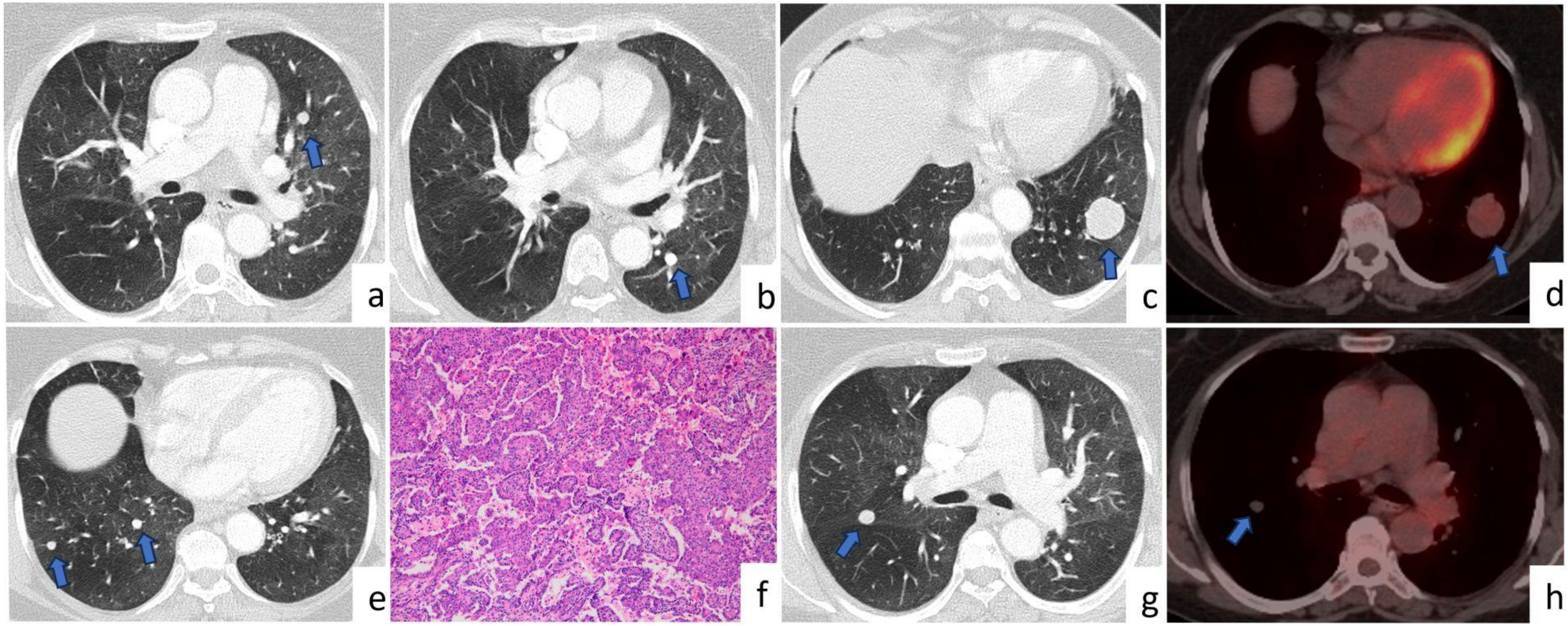

Fig. S2. Pulmonary sclerosing pneumocytoma. A 56-year-old female patient with incidentally detected multiple lung nodules. Axial CT images **(a- c, e, g)**, show bilateral and multiple solid nodules ranging in size **(arrows)**. There is no apparent FDG uptake on PET-CT images **(d, h) (arrows)**. Photomicrograph shows a papillary growth pattern with two cell types: cuboidal surface cells and round stromal cells (H&Ex100) **(f).**
